# Microbial metabolism mediates interactions between dissolved organic matter and clay minerals in streamwater

**DOI:** 10.1038/srep30971

**Published:** 2016-08-02

**Authors:** W. R. Hunter, T. J. Battin

**Affiliations:** 1 Queen’s University Marine Laboratory, School of Biological Sciences, The Queen’s University of Belfast, Portaferry, BT22 1PF, United Kingdom; 2 Stream Biofilm and Ecosystem Research Laboratory, School of Architecture, Civil and Environmental Engineering, Ecole Polytechnique Fédérale Lausanne (EPFL), CH-1015 Lausanne, Switzerland

## Abstract

Sorption of organic molecules to mineral surfaces is an important control upon the aquatic carbon (C) cycle. Organo-mineral interactions are known to regulate the transport and burial of C within inland waters, yet the mechanisms that underlie these processes are poorly constrained. Streamwater contains a complex and dynamic mix of dissolved organic compounds that coexists with a range of organic and inorganic particles and microorganisms. To test how microbial metabolism and organo-mineral complexation alter amino acid and organic carbon fluxes we experimented with ^13^C-labelled amino acids and two common clay minerals (kaolinite and montmorillonite). The addition of ^13^C-labelled amino acids stimulated increased microbial activity. Amino acids were preferentially mineralized by the microbial community, concomitant with the leaching of other (non-labelled) dissolved organic molecules that were removed from solution by clay-mediated processes. We propose that microbial processes mediate the formation of organo-mineral particles in streamwater, with potential implications for the biochemical composition of organic matter transported through and buried within fluvial environments.

In fluvial environments, amino acids and other dissolved organic compounds coexist with a range of organic and inorganic particles, onto which they can adsorb and desorb^1^,^2^. Amino acids are important constituents of the pool of dissolved organic matter (DOM) in aquatic ecosystems. They are prime sources of organic carbon and nitrogen for the microbial metabolism^3^,^4^ and they may serve as olfactory cues mediating animal behaviour in streams and rivers^3^,^4^,^5^. The fine-scale mechanisms that control the persistence of amino acids in streams and rivers, however, remains poorly understood. Organo-mineral complexation is potentially an important control upon the bioavailability of amino acids and other dissolved organic compounds. Organo-mineral complexes form via ion exchange reactions between dissolved organic molecules and the mineral surfaces^1^,^2^,^6^,^7^. Within these reactions, mineral structure is critical, such that the potential for stabilisation of organic molecules against microbial degradation is believed to be an important property of clay minerals^1^,^7^,^8^,^9^,^10^. Kaolinite and montmorillonite are common clay species in soils and sediments^11^, and they highlight the structural variability of these minerals. Kaolinite is composed of a single tetrahedral sheet and a single octahedral sheet held together by hydrogen bonding of aluminol (Al–OH) and siloxane (Si–O) groups. These bonding forces mean that kaolinite is a non-swelling clay with limited cation exchange capacity^11^,^12^,^13^. Montmorillonite is composed of an octahedral sheet of alumina sandwiched between two tetrahedral silicate sheets. These sheets are only weakly bonded and so can become separated by interactions with water (as a polar liquid), providing a greater surface area to which dissolved organic molecules may bind^6^,^12^,^13^. Mineral structure and the ambient chemical environment are important properties determining the sorption dynamics of organic molecules to minerals. For instance, it has been shown that the more complex structure of montmorillonite binds more amino acids under abiotic conditions^1^,^7^,^8^,^9^. The role of microbial activity for organo-mineral interactions and resulting biogeochemical fluxes in aquatic ecosystems are currently poorly understood^10^. Fine-scale mechanistic understanding of these processes is critical though to predict the dynamics of minerals and DOM, and their link to the carbon cycle in streams and rivers^14^.

In this study we test how the competing processes of microbial metabolism and organo-mineral complexation affect the fate of free amino acids and other dissolved organic molecules in streamwater. We amended natural streamwater microbial communities with ^13^C-labelled amino acids and exposed them to different concentrations of initially organic-free kaolinite and montmorillonite particles as outlined in Fig. 1. By tracing the ^13^C label in the organic and inorganic carbon pools allowed us to assess the mineralization and retention of the amino acids. Loss of amino acids and DOM was then estimated via mass balance, from which we infer the relative importance of organo-mineral interactions for carbon fluxes in our experimental systems. Organo-mineral sorption is a surface-area limited reaction and we hypothesize that clay type and concentration influence the sorption dynamics of free amino acids and their mineralization by microorganisms.

## Results

### Microbial respiration

**Figure 2:**
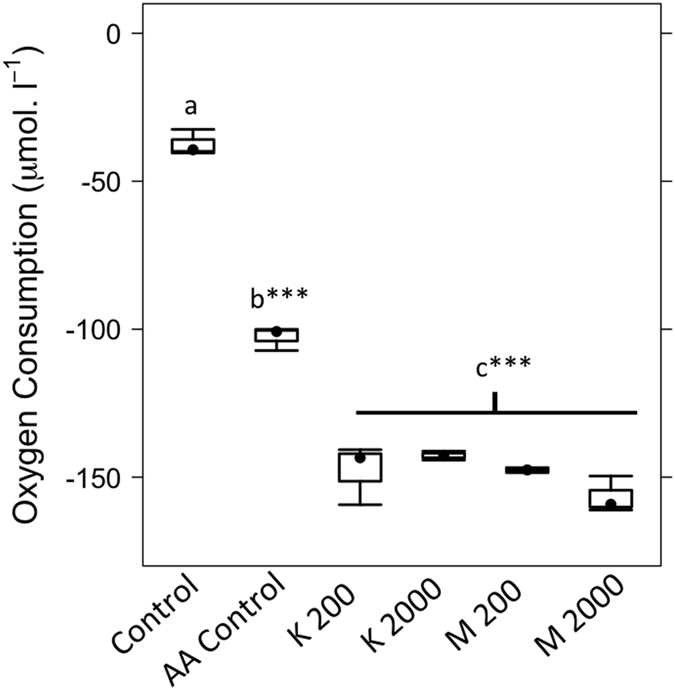
Microbial respiration in the experimental systems. Boxplots of oxygen consumption over the 72-hour incubations. Letters indicate treatments that are statistically indistinguishable based on post-hoc Tukey’s HSD tests (p > 0.05). Significant differences between treatments are identified at p < 0.05 (*); p < 0.01 (**) or p < 0.001 (***).

The experimental addition of the ^13^C-labelled amino acids and clay particles to natural microbial communities in streamwater had significant effects upon microbial oxygen consumption (p < 0.001, F = 214.5; df = 5) (Fig. 2). Addition of the ^13^C-labelled amino acids significantly increased oxygen consumption between the two controls (p < 0.001), and the presence of clay particles significantly increased respiration relative to the amino acid control (p < 0.001). There were no significant differences in oxygen consumption observed between the four clay treatments (Fig. 2).

### Fluxes of the ^13^C-labelled amino acids and non-labelled DOM

**Figure 3:**
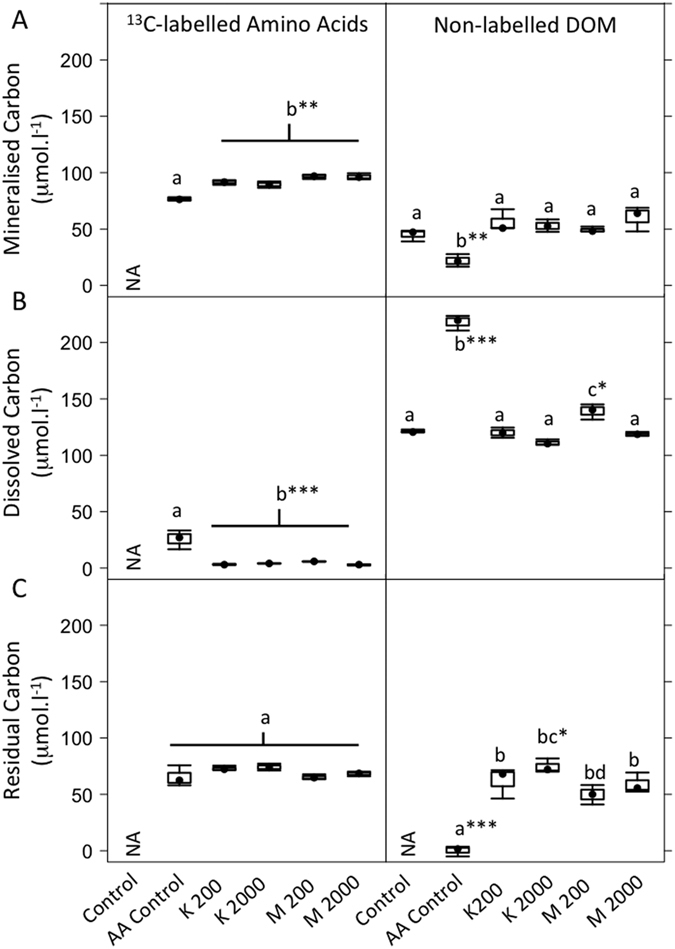
Fluxes of the ^13^C-labelled amino acids and non-labelled dissolved organic carbon. (**A**) Mineralisation of the amino acids and other DOM, (**B**) their concentrations remaining in the DOC pool and (**C**) the residual amino acid and non-labelled DOM concentrations (not detected in the DIC or DOC) after 72 hour incubations. Letters indicate treatments that are statistically indistinguishable based on post-hoc Tukey’s HSD tests (p > 0.05). Significant differences are between treatments are identified at p < 0.05 (*); p < 0.01 (**) or p < 0.001 (***).

Upon addition of the clay particles we observed a significant increase in the mineralisation of both the ^13^C-labelled amino acids (p < 0.001, F = 38.63; df = 4) and non-labelled DOM within streamwater (p = 0.001, F = 10.96; df = 5) (Fig. 3A). This reflects the increased oxygen demand observed in Fig. 2, with no significant differences between clay treatments.

The presence of clay minerals significantly reduced the concentrations of dissolved ^13^C-labelled amino acids (p < 0.001, F = 19.85; df = 4) and non-labelled DOM (p < 0.001, F = 234.40; df = 5) (Fig. 3B). There were no significant differences in dissolved ^13^C-labelled amino acids between clay treatments. However, non-labelled DOM concentrations showed a treatment effect, with significantly higher DOC concentrations recorded in the 200 mg l^−1^ montmorillonite treatment, compared with the other clay treatments (Fig. 3B).

The residual pool of ^13^C-labelled amino acids was calculated as the proportion of the ^13^C-amino acid not detected in either the DIC or DOC (Fig. 3C). The residual ^13^C-labelled amino acids exhibited no difference between the clay treatments and the control (p = 0.12, F = 2.39; df = 4). A residual non-labelled carbon pool was calculated as the proportion non-labelled DOM not detected in either the DIC or DOC of the clay treatments, relative to the amino acid control. In the presence of clay particles there was a significant loss of non-labelled DOM to the residual pool (p < 0.001, F = 62.61, df = 4), with no significant differences between the clay treatments (Fig. 3C).

### Changes in non-labelled DOM concentrations

**Figure 4:**
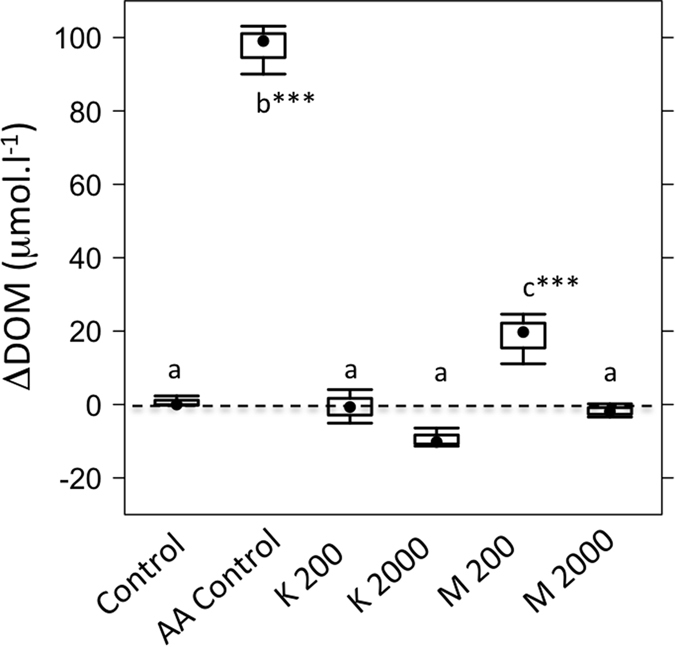
Changes in non-labelled DOM concentrations relative to background DOC concentrations (120.55 ± 0.17 μmol l^−1^), over the 72-hour incubations. Letters indicate treatments that are statistically indistinguishable based on post-hoc Tukey’s HSD tests (p > 0.05). Significant differences are between treatments are identified at p < 0.05 (*); p < 0.01 (**) or p < 0.001 (***).

The change in the non-labelled DOM concentrations (ΔDOM) was calculated relative to background streamwater DOC concentration. These data were corrected against the final concentration of dissolved ^13^C-labelled amino acids and reveal significant DOM production in the amino acid controls (p < 0.001, F = 234.4, df = 5) (Fig. 4). The ΔDOM values of the clay treatments were comparable with the procedural control.

## Discussion

In this study we tested how changes in clay type (kaolinite or montmorillonite) and concentration (0, 200 or 2000 mg l^−1^) influence the sorption dynamics of dissolved amino acids and their mineralization by microorganisms, in streamwater. The study suggests that microbial metabolism was the dominant process determining the fate of dissolved amino acids, with 55.7 ± 2.3% of the amino acids mineralised in the clay treatments and 45.7 ± 0.9% mineralised in the amino acid control (Fig. 3A). The addition of ^13^C-labelled amino acids appears to stimulate microbial activity, with a concomitant increase in the production of non-labelled DOM. This is observable in the amino acid control, however, there was no increase in non-labelled DOM concentrations. This suggests that the presence of clay minerals within the streamwater facilitated the removal of non-labelled DOM. The incubations were limited to 72 hours, over which time dissolved oxygen concentrations in the clay treatments decreased by ~50%. This reflects the time-scales over which previous studies report amino acid sorption to occur^1^,^2^ and mitigates against potentially confounding effects associated with the switch between aerobic and anaerobic metabolism by the streamwater microbial community. However, we acknowledge that the short duration of these experiments limit our capacity to discuss the longer term preservation of carbon within organo-mineral complexes.

Previous studies have focussed on the organo-mineral interactions, both in artificial^1^,^6^ and natural systems^2^, yet microbial contributions to these processes remain understudied. Amino acids readily adsorb to mineral particles, but often this does not offer complete protection from microbial degradation. Instead a portion of the sorbed amino acids remains available for the microbial metabolism^9^,^10^,^15^. In aquatic ecosystems, the interplay between clay particles and microorganisms may facilitate the flocculation of dissolved organic matter^16^,^17^,^18^. This process entails the concentration of active microbial cells and dissolved organic matter around a mineral core, which can be sites of elevated microbial activity in stream ecosystems^19^. DOM molecules adsorbed to minerals may thus be brought into close proximity to microorganisms, and the metabolic activity of microorganisms will impact both flocculation and adhesion of DOM. In turn, this may change the chemical composition of organo-mineral particles and their dynamics as they move through the fluvial continuum^19^,^20^.

Clay minerals often enter fluvial networks via headwater streams where steep hill slopes and loose parent material facilitate erosion^21^. Concentrations of clay minerals used in this study were high, but reflect the sediment loads in streams during storm events^22^,^23^,^24^. During these events, the adsorption of DOC molecules to clay minerals metabolism may compete with microbial heterotrophs during phases of reduced DOC availability. In our experimental systems, microbial activity was the primary control on the fate of the ^13^C-labelled amino acids. This underscores the role of amino acids as a source of carbon and nitrogen for microbial metabolism^3^,^4^, and that they are preferentially metabolised relative to other DOC sources^25^.

**Figure 5:**
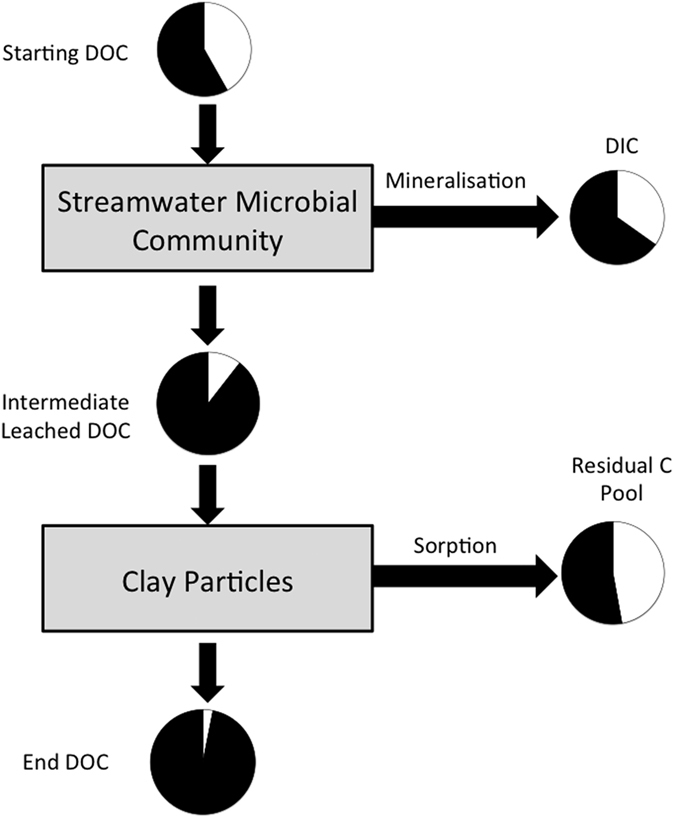
Conceptual diagram of the proposed pathways for microbially mediated organo-mineral sorption within the flask experiments. Here we show the proportion of ^13^C-labelled amino acids (black) and non-labelled DOM (white) within the DOC, DIC and Residual C pools. Intermediate Leached DOC proportions are derived from the proportions of ^13^C-labelled amino acids and non-labelled DOM in the DOC of Amino Acid Control treatment (no clay).

We experimentally demonstrate that the addition of amino acids stimulated DOC production by the microbial community (Fig. 4). This supports the notion that microorganisms simultaneously consume and produce dissolved organic matter^26^. However, in the presence of clay particles, there was comparatively little change in the non-labelled DOM pool within the experiment. Given that microbial respiration and amino acid mineralisation both increased in response to clay addition, we would predict non-labelled DOM production to likewise increase. Within the clay treatments, there was little increase of non-labelled DOM (Figs 3B and 4), suggesting the removal of the molecules via organo-mineral sorption (Figs 3C and 4). We propose that microorganisms mediate organo-mineral sorption in aquatic systems. This occurs via preferential mineralisation of labile organic molecules, such as amino acids, leading to the release of non-characterised dissolved organic molecules. These are in turn removed from solution via organo-mineral sorption (Fig. 5). The amino acid doses used within this study were high, reflecting the maximal DOC concentrations detected within our study system (the Oberer Seebach stream). This represents a standard practise in stable-isotope labelling experiments, to ensure the ^13^C-label is detectable above the natural variations in the δ^13^C-signature of each biochemical pool^10^,^27^. Further study is necessary to fully elucidate how microbial activity influences organo-mineral sorption at natural streamwater DOM concentrations. Here we provide a data-driven hypothesis and conceptual model (Fig. 5) that will support future work on this important biogeochemical question.

Finally, there were no clear trends in the fate of either ^13^C-labelled amino acids or non-labelled DOM associated with clay type (kaolinite or montmorillonite) or concentration (200 or 2000 mg l^−1^). The clay concentrations used in the present study were high, reflecting point source measurements of suspended sediments associated with storm events and mining impacts in high-order streams^22^,^23^,^24^. However, it is likely that the effects of microbial metabolism combined with these high clay concentrations may have obscured the more subtle effects of clay structure upon organo-mineral interactions^1^. Based upon the structural differences between the two clays, we suggest montmorillonite to exhibit a higher carrying capacity for, and stronger sorption of organic molecules^6^,^12^. However, this is not reflected within our results (Fig. 3). Based on the data available it is not possible to draw any sound conclusion upon theses fine-scale processes governing organo-mineral sorption. To fully elucidate these mechanisms would require further experimentation, investigating organo-mineral interactions across a range of clay doses under both biotic and abiotic conditions. Whilst this represents a clear avenue for future study, it does not invalidate our central observation that organo-mineral interactions were governed by microbial metabolic processes, in this study.

## Material and Methods

Experiments were conducted over 72 hours (10 °C, darkness) in pre-combusted 100 mL Schott bottles with no headspace. We used raw streamwater from Oberer Seebach (OSB, Lunz am See, Austria) free of coarse particles (larger than 63 μm) but that contained the natural microbial community (up to 6 × 10^9^ cells liter^−1^). Experiments were conducted in triplicate bottles amended with a 167 μmol C l^−1^ of 10 atom % ^13^C-labelled “cell-free” amino acid mix (Euriso-Top GmbH, Germany) and either 0, 200 or 2000 mg l^−1^ of kaolinite or montmorillonite (Sigma-Aldrich, UK). Clay concentrations were 10- and 100- times the maximal suspended sediment loads of OSB. Triplicate procedural (no clay, no ^13^C-labelled amino acids) and amino acid (no clay) controls were established alongside the experimental treatments. In each bottle, oxygen consumption by microorganisms was determined from measurements if dissolved oxygen concentration using a PreSens Oxy-4 optode system (PreSens Precision Sensing Gmbh, Germany).

Concentrations and isotopic ratios of DOC and DIC were analysed in the streamwater (as background) and in the assay bottles. DOC concentrations and ^12^C/^13^C ratios were analysed by liquid chromatography isotope ratio mass spectroscopy (LC-IRMS) using a Dionex High Precision Liquid Chromatograph (Dionex Corporation, Sunnyvale, CA, USA) coupled to a Finnigan Delta V Advantage isotope ratio mass spectrometer via a Finnigan LC Isolink Interface (Thermo Fisher, Bremen, Germany). Analyses were performed on 0.5 ml filtered (Whatman GF/F, 0.7 μm) water samples acidified with 10 ml of 85% othophosphoric acid^28^. Concentrations and ^12^C/^13^C ratios ratios of DIC were determined from 1 ml water samples, injected into N_2_-flushed 12 ml vials containing 1 ml 85% orthophosphoric acid to liberate gaseous CO_2_ into the headspace. Headspace gas was then analysed via purge-and-trap isotope ratio mass spectrometry (PT-IRMS) using a GasBench II gas preparation unit coupled to a Delta V Advantage IRMS (Thermo Fisher, Bremen, Germany)^27^. All samples were processed within 10 days of sampling. Background DOC and DIC concentrations in the streamwater averaged 120.55 ± 0.17 μmol l^−1^ and 2509.9 ± 52.81 μmol l^−1^ at the beginning of the experimental incubations.

The ^13^C enrichment of DOC and DIC samples was determined from the ^12^C/^13^C ratios by


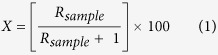


where *X* is the ^13^C enrichment (in atom %) and *R*_*sample*_ is the ^12^C/^13^C ratio of the sample. We then calculated the concentration of ^13^C of each sample (*E*) as


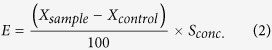


where *X*_*sample*_is the ^13^C enrichment of the sample (atom %), *X*_*control*_is the background ^13^C value (atom %) and *S*_*conc.*_ is the concentration. Contributions of ^13^C-labelled amino acids to the DIC and DOC pools were determined by normalising the ^13^C concentrations to the specific labelling of the ^13^C-labelled amino acids (10 atom %). The contributions of non-labelled DOM to the DOC pools were calculated as the differences between total DOC and dissolved ^13^C-labelled amino acids. The contribution of respired non-labelled DOM to the DIC pool was calculated from the measured changes in oxygen concentration and the ^13^C-labelled amino acids contributions to the DIC pool as


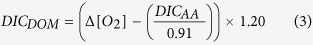


where Δ[O_2_] is the measured change in oxygen concentration, DIC_AA_ is the contribution of the ^13^C-labelled amino acids to the DIC pool, 0.91 is the respiratory quotient calculated for the ^13^C-labelled amino acids (supplementary Table 1) and 1.20 is the Berggern *et al.*’s^29^ general respiratory quotient for aquatic bacterioplankton utilising an unknown DOC source. The residual pools of ^13^C- labeled amino acids and non-labelled DOM were calculated as the proportion of each not detected in either the DIC or DOC. All data are summarised in Supplementary Tables 2 and 3.

We used a one-way factorial design to test for significant differences in oxygen consumption, and the contributions of ^13^C-labelled amino acids and non-labelled DOM to the DIC, DOC and residual C pools. All data were visually explored prior to analysis to ensure their conformity to assumptions of normality and homoscedacity^30^. Differences between treatments were tested using a one-way analysis of variance (ANOVA). Pairwise comparisons were done using the Tukey’s Honest Significant Difference. Data analyses were carried out using the *base* and *lattice* packages in R^31^,^32^.

## Additional Information

**How to cite this article**: Hunter, W. R. and Battin, T. J. Microbial metabolism mediates interactions between dissolved organic matter and clay minerals in streamwater. *Sci. Rep.* **6**, 30971; doi: 10.1038/srep30971 (2016).

## Supplementary Material

Supplementary Information

## Figures and Tables

**Figure 1:**
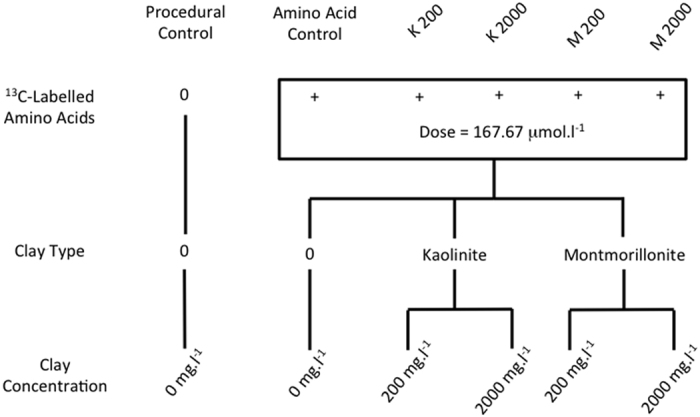
Experimental design matrix. Illustration of the experimental design, with each treatment was replicated in triplicate independent systems. As the experimental design was not fully factorial, statistical analyses were done by one-way analysis of variance.
