# Supplementary material for: Microbial metabolism mediates interactions between dissolved organic matter and clay minerals in streamwater
Source: Sci Rep. 2016 Aug 2;6:30971. doi: 10.1038/srep30971 (PMC4969585; doi:10.1038/srep30971)
Supplement: Supplementary Information [file srep30971-s1.doc]

# Microbial metabolism mediates interactions between dissolved organic matter and clay minerals in streamwater

Hunter, W.R.^*^; Battin, T.J.^*^

*Corresponding Author.

**Supplementary Information**

**Supplementary Table 1. Calculated respiratory quotients for the 21 ^13^C-labelled amino acids, and the overall respiratory quotient for the mixture.**

| Amino Acid Proportion Calculated Proportional  of Mix RQ RQ |
| --- |
| Asp 0.038 1.125 0.042  Glu 0.042 0.900 0.038  Asn 0.037 0.750 0.028  Ser 0.036 1.170 0.042  Gln 0.042 0.900 0.038  His 0.048 0.830 0.040  Gly 0.033 0.250 0.008  Thr 0.042 0.250 0.010  Ala 0.042 0.500 0.021  Arg 0.042 0.920 0.039  Tyr 0.062 1.220 0.075  Val 0.053 1.200 0.064  Met 0.042 0.800 0.033  Trp 0.067 1.040 0.070  Phe 0.067 0.890 0.060  Ile 0.056 1.125 0.063  Leu 0.056 1.125 0.063  Lys 0.050 1.167 0.059  Pro 0.053 1.100 0.058  Cys 0.031 0.333 0.010  Cystine 0.061 0.750 0.045    **Overall AA RQ = 0.907** |

**Supplementary Table 2.** Summary of the dissolved inorganic carbon data, oxygen consumption data (Δ[O_2_]) and calculated values of ^13^C-labelled Amino acids (AA) and non-labelled DOM respired within the experiments.

| Treatment Conc. DIC δ^13^C DIC Δ[O_2_] Conc DI^13^C AA respired DOM respired  (μmol.l^-1^) (‰) (μmol.l^-1^) (μmol.l^-1^) (μmol.l^-1^) (μmol.l^-1^) |
| --- |
| Procedural Control 2594.585 -8.227 32.468 0.000 0.000 38.962  (Streamwater) 2499.318 -8.190 39.347 0.000 0.000 47.216  2570.513 -8.107 40.441 0.000 0.000 48.529  Amino Acid Control 2607.633 256.480 100.785 7.525 75.253 21.379  (Streamwater + _f_AAs) 2744.291 253.193 100.044 7.822 78.217 16.569  2688.829 252.025 107.218 7.629 76.295 27.721  Kaolinite 200 mg.l^-1^ 3684.931 219.877 143.430 9.167 91.673 50.829  2807.346 283.344 140.728 8.921 89.214 50.839  2885.875 288.751 159.320 9.341 93.405 67.605  Montmorillonite 2895.844 290.737 147.557 9.435 94.353 52.235  200 mg.l^-1^ 2888.692 299.784 146.785 9.696 96.959 47.862  2923.778 300.124 148.464 9.824 98.245 48.174  Kaolinite 2000 mg.l^-1^ 3434.545 231.056 142.609 8.962 89.621 52.558  2983.549 257.932 144.230 8.657 86.573 58.536  2985.166 275.047 141.206 9.217 92.174 47.497  Montmorillonite 3189.572 262.413 161.104 9.411 94.105 68.819  2000 mg.l^-1^ 3101.571 286.089 149.639 9.949 99.490 47.938  2676.101 321.032 159.169 9.600 95.998 63.993 |

**Supplementary Table 3.** Summary of the dissolved organic carbon carbon data and calculated values for the dissolved and residual ^13^C-labelled Amino acids (AA) and non-labelled DOM concentrations within the experiments.

| Treatment DOC δ^13^C DOC DO^13^C AA conc. AA residual DOM conc. DOM residual  (μmol.l^-1^) (μmol.l^-1^) (μmol.l^-1^) (μmol.l^-1^) (μmol.l^-1^) (μmol.l^-1^) |
| --- |
| Procedural Control 122.887 -17.604 0.000 0.000 0.000 122.887 NA  (Streamwater) 120.390 -15.305 0.000 0.000 0.000 120.390 NA  120.556 -9.454 0.000 0.000 0.000 120.556 NA  Amino Acid Control 240.280 623.369 1.664 16.637 75.789 223.643 0.000  (Streamwater + _f_AAs) 246.607 997.657 2.699 26.989 62.474 219.618 3.655  243.943 1253.079 3.334 33.344 58.041 210.599 1.523  Kaolinite 200 mg.l^-1^ 119.224 197.851 0.289 2.891 73.116 116.333 72.681  122.887 210.448 0.301 3.011 75.454 119.876 69.127  127.383 185.254 0.277 2.772 71.503 124.611 47.627  Montmorillonite 150.695 322.801 0.553 5.533 67.794 145.162 42.446  200 mg.l^-1^ 146.199 356.159 0.590 5.897 64.824 140.303 51.679  137.707 389.518 0.605 6.053 63.382 131.654 60.014  Kaolinite 2000 mg.l^-1^ 118.225 302.771 0.408 4.083 73.975 114.141 73.144  114.229 296.828 0.387 3.872 77.235 110.357 70.950  113.563 337.933 0.436 4.356 71.150 109.207 83.139  Montmorillonite 120.556 245.520 0.341 3.414 70.161 117.142 53.881  2000 mg.l^-1^ 122.887 147.096 0.216 2.163 66.027 120.724 71.181  121.722 216.000 0.306 3.056 68.625 118.666 57.185 |
